# The *LRRK2* R1441G+M1646T haplotype is associated with slower motor symptom progression in Parkinson’s disease

**DOI:** 10.64898/2026.08.26.26361428

**Published:** 2026-08-31

**Authors:** Jackson G. Schumacher, Xinyuan Zhang, Jian Wang, Xiqun Chen

## Abstract

**Background:**

Mutations in leucine-rich repeat kinase 2 ( *LRRK2* ) are the most common genetic risk factor for Parkinson’s disease (PD). G2019S, the most common pathogenic variant, has been linked to milder motor symptoms, but the effects of other LRRK2 variants on disease trajectory remain incompletely characterized. R1441G, the second most common pathogenic variant, co-occurs with the PD risk variant M1646T on a shared haplotype. Whether this haplotype confers a distinct rate of motor progression has not been established.

**Methods:**

We analyzed up to 12 years of longitudinal data from 603 participants in the Parkinson’s Progression Markers Initiative (PPMI) with PD and available whole-genome sequencing data: 394 sporadic PD, 169 G2019S carriers, 20 R1441G+M1646T carriers, and 20 M1646T carriers. Motor symptom progression (MDS-UPDRS III) was assessed using linear mixed-effects models with genotype-by-time interactions, adjusted for age at onset, disease duration at baseline, sex, race, baseline score, and levodopa equivalent daily dose.

**Results:**

R1441G+M1646T carriers exhibited 76% slower progression in OFF-state MDS-UPDRS III than sporadic PD (0.50 vs. 2.04 points/year; β=−1.54 [95% CI: −2.48, −0.60]; p=0.001). G2019S carriers exhibited 26% slower progression (1.52 points/year; β=−0.52 [−0.99, −0.06]; p=0.03). M1646T carriers did not differ from sporadic PD (p=0.60). Slower progression in R1441G+M1646T carriers was characterized by attenuated bradykinesia (64% slower; p=0.008), axial decline (76% slower; p=0.002), and a lack of orofacial symptom progression (p<0.001). R1441G+M1646T carriers also exhibited 55% slower self-reported motor decline (MDS-UPDRS II; p=0.04)

**Conclusions:**

R1441G+M1646T carriers exhibit substantially slower motor progression than sporadic PD while M1646T carriers do not.

## Full Text Availability

The license terms selected by the author(s) for this preprint version do not permit archiving in PMC. The full text is available from the preprint server.

